# Evaluating Humoral Immunity Elicited by XBB.1.5 Monovalent COVID-19 Vaccine

**DOI:** 10.3201/eid3006.240051

**Published:** 2024-06

**Authors:** Xammy Huu Wrynla, Timothy A. Bates, Mila Trank-Greene, Mastura Wahedi, Fikadu G. Tafesse, Marcel El Curlin

**Affiliations:** Oregon Health and Science University, Portland, Oregon, USA

**Keywords:** COVID-19, respiratory infections, severe acute respiratory syndrome coronavirus 2, SARS-CoV-2, SARS, coronavirus disease, viruses, coronavirus, vaccines, allergy and immunology, antibodies, United States

## Abstract

Because novel SARS-CoV-2 variants continue to emerge, immunogenicity of XBB.1.5 monovalent vaccines against live clinical isolates needs to be evaluated. We report boosting of IgG (2.1×), IgA (1.5×), and total IgG/A/M (1.7×) targeting the spike receptor-binding domain and neutralizing titers against WA1 (2.2×), XBB.1.5 (7.4×), EG.5.1 (10.5×), and JN.1 (4.7×) variants.

A monovalent COVID-19 vaccine containing the XBB.1.5 variant SARS-CoV-2 spike protein was approved in September 2023, but uptake has been hesitant (*1*). Evaluating the immunogenicity of variant-adapted vaccines could inspire trust in COVID-19 immunization, especially as neutralization-evading variants such as JN.1 emerge. Studies have demonstrated induction of antibodies capable of neutralizing variant spike proteins (*2*–*4*), but those studies used pseudo-typed virus that recombinantly expressed variant spike proteins, not true SARS-CoV-2. We evaluated immunogenicity of XBB.1.5 vaccination in humans by using live SARS-CoV-2 clinical isolates to capture the biology of virus neutralization.

During October–November 2023, we recruited healthcare workers at Oregon Health and Science University (OHSU) in Portland, Oregon, USA. We collected paired serum samples from participants: 1 on the day XBB.1.5 monovalent booster vaccine (Moderna, https://www.modernatx.com) was administered, and 1 ≈21 days after vaccination. To identify recent infection, we used ELISA to detect nucleocapsid antibodies. We used 50% ELISA effective concentrations to determine IgG, IgA, IgM, and total IgG/A/M titers against the ancestral spike receptor binding domain (RBD) (Appendix). We determined SARS-CoV-2 neutralizing antibody titers by using 50% focus reduction neutralization tests against the ancestral (wild-type) strain and XBB.1.5, EG.5.1, and JN.1 variants (Figure, panel A). We used restricted effect maximum-likelihood model or repeated analysis of variance measures with Šídák’s multiple comparison tests to calculate p values and considered p<0.05 statistically significant. The OHSU institutional review board approved this study, and participants provided written informed consent. 

**Figure:**
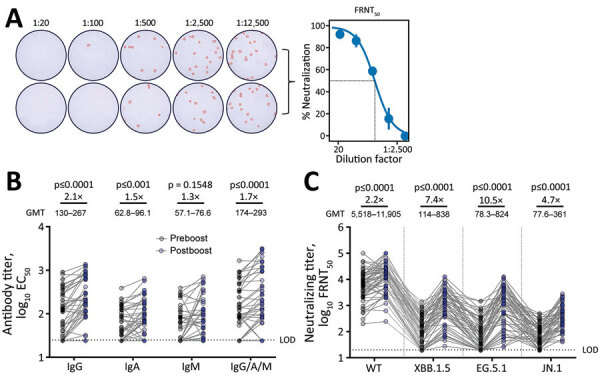
SARS-CoV-2 antibody titers in an evaluation of humoral immunity elicited by XBB.1.5 monovalent COVID-19 vaccine. A) Duplicate wells infected with live SARS-CoV-2 virus at serially diluted titers. OD was measured at 492 nm using a CLARIOstar plate reader (BMG LABTECH, https://www.bmglabtech.com). Wells were stained and counted to create representative FRNT_50_ curve at right. B) Preboost and postboost serum antibody isotype titers against spike RBD. C) Neutralizing titers against live ancestral (WT) SARS-CoV-2 and variants. GMT for each bar was calculated in Prism (GraphPad Software Inc., https://www.graphpad.com). All individual data points are displayed as filled circles. Boost ratios were calculated by dividing the post-XBB.1.5 vaccination GMT (postboost) by pre-vaccination GMT (preboost). Reported p values were calculated using restricted effect maximum-likelihood model (B) or 1-way repeated measures analysis of variance (C) with Šídák's multiple comparisons tests. EC_50_, 50% ELISA effective concentration; FRNT_50_, 50% focus reduction neutralization; GMT, geometric mean titer; LOD, lower limit of detection; OD, optical density; WT, wild-type.

We enrolled 55 participants, 37 (67%) female and 18 (33%) male; mean age was 53 years. Eleven (20%) preboost samples and 15 (27%) postboost samples were positive for nucleocapsid antibodies. We included those samples to demonstrate generalized boosting in a population with heterogenous exposure history; however, removing those participants from analysis resulted in similar antibody induction by XBB.1.5 vaccination (Appendix Figure, panels A, B). The XBB.1.5 vaccine boosted total serum spike RBD–specific IgG/A/M; after boosting, geometric mean titers (GMT) rose 1.7-fold from 293 (95% CI 195–442) to 174 (95% CI 124–244) (p<0.0001). IgG isotypes demonstrated a greater increase than IgA isotypes (IgG postboost GMT 267 [95% CI 196–363], preboost GMT 130 [95% CI 95.7–176], a 2.1-fold change [p<0.0001]; IgA postboost GMT 96.1 [95% CI 74.6–124], preboost GMT 62.8 [95% CI 50.3–78.3], a 1.5-fold change [p = 0.0002]). The reason for this difference is unclear. IgM isotypes trended toward a slight increase, likely because IgM is short-lived; postboost GMT was 76.6 (95% CI 57.6–102) versus preboost GMT 57.1 (95% CI 44.5–73.2), a 1.3-fold change (p = 0.1548) (Figure, panel B). Of note, the XBB.1.5 vaccine boosted neutralizing titers against the wild-type strain; postboost GMT was 11,905 (95% CI 8,454–16,766) versus preboost GMT 5,518 (95% CI 3,899–7,809), a 2.1-fold change (p<0.0001). The vaccine also boosted neutralizing titers against the vaccine-matched XBB.1.5 variant; postboost GMT was 838 (95% CI 548–1,281) versus preboost GMT 114 (95% CI 80.9–162), a 7.4-fold change (p<0.0001). In addition, the vaccine boosted neutralizing titers against EG.5.1 by 10.5 fold (postboost GMT 824 [95% CI 518–1,311] vs. preboost GMT 78.3 [95% CI 55.0–112]; (p<0.0001), and the JN.1 variant by 4.7 fold (postboost GMT 361 [95% CI 270–483] vs. preboost GMT 77.6 [95% CI 60.7–99.2]; p<0.0001) (Figure, panel C).

To assess changes in the proportion of serum antibodies with neutralizing capacity, we divided the serum neutralizing titer against each variant by the total IgG/A/M titer to produce a neutralizing potency index (NPI). The NPI against wild-type strain was unchanged by XBB.1.5 monovalent vaccination. That finding is likely explained by preexisting neutralizing immunity that is dominated by responses against ancestral epitopes. However, the XBB.1.5 vaccine elicited an NPI increase against XBB.1.5, EG.5.1, and JN.1 variants (Appendix Figure, panel C).

Our results demonstrated that, before vaccination, persons have low titers of antibodies capable of neutralizing XBB.1.5, EG.5.1, and JN.1, and that the XBB.1.5 monovalent vaccine increases the capacity of serum antibodies to neutralize contemporary variants. IgG, IgA, and total IgG/A/M titers were boosted, which likely includes expansion of a nonneutralizing compartment that may influence disease severity and longer-term protection through Fc effector functions (*5*). Indeed, the XBB.1.5 monovalent vaccine was reported to reduce risk for COVID-19 hospitalization by 76.1% in Denmark (*6*). In the United States, an analysis of 2 vaccine effectiveness data networks estimated 52% (95% CI 47%–57%) and 43% (27%–56%) vaccine effectiveness against hospitalization (*7*). 

In summary, these data provide direct evidence for immunogenicity of XBB.1.5 monovalent vaccines against SARS-CoV-2 variants and supports public health recommendations to stay current with adapted COVID-19 vaccines. Neutralizing antibodies were boosted against the wild-type, vaccine-matched, and emergent strains, suggesting that updated vaccines enhance protection against infection by historic and novel variants.

Appendix Additional information on an evaluation of humoral immunity elicited by XBB.1.5 monovalent COVID-19 vaccine.
