# Correction: Vol. 30, No. 6

**DOI:** 10.3201/eid3107.C13107

**Published:** 2025-07

**Keywords:** errata, erratum, correction

The first author’s name has changed in Evaluating Humoral Immunity Elicited by XBB.1.5 Monovalent COVID-19 Vaccine (X.H. Wrynla et al.). The article has been corrected online (https://wwwnc.cdc.gov/eid/article/30/6/24-0051_article).

